# Sphingolipids and Lymphomas: A Double-Edged Sword

**DOI:** 10.3390/cancers14092051

**Published:** 2022-04-19

**Authors:** Alfredo Pherez-Farah, Rosa del Carmen López-Sánchez, Luis Mario Villela-Martínez, Rocío Ortiz-López, Brady E. Beltrán, José Ascención Hernández-Hernández

**Affiliations:** 1 Tecnologico de Monterrey, Escuela de Medicina y Ciencias de la Salud, Monterrey 64710, Nuevo Leon, Mexico; frephe@tec.mx (A.P.-F.); lopezsanchezr@tec.mx (R.d.C.L.-S.); rortizl@tec.mx (R.O.-L.); 2 Facultad de Medicina, Universidad Autónoma de Sinaloa, Culiacán Rosales 80030, Sinaloa, Mexico; l.villela@isssteson.gob.mx; 3 Hospital Fernando Ocaranza, ISSSTE, Hermosillo 83190, Sonora, Mexico; 4 Centro Médico Dr. Ignacio Chávez, ISSSTESON, Hermosillo 83000, Sonora, Mexico; 5 Hospital Edgardo Rebagliati Martins, Lima 15072, Peru; beltrang@usmp.pe; 6 Instituto de Investigaciones en Ciencias Biomédicas, Universidad Ricardo Palma, Lima 1801, Peru

**Keywords:** lymphoma, cancer, lipid metabolism, lipidomics, sphingolipids

## Abstract

**Simple Summary:**

Cancer is an incredibly smart disease, so much so that it can entirely reprogram our cells’ metabolism to ensure its survival and dissemination. Lymphomas are a particularly interesting group of cancers because they are very diverse and exhibit complex pathogenic mechanisms that can make them clinically challenging. Many of these mechanisms involve the dysregulation of a biologically relevant family of lipids known as sphingolipids. These molecules are involved in virtually every cellular process and therefore studying them in this context is crucial. However, sphingolipid biochemistry is intricate and the synergistic and antagonic effects of different sphingolipid species with one another has long puzzled scientists. This duality is also observed in cancer, but research specifically focusing on lymphomas is limited. Could novel biomarkers and therapies against lymphomas be hiding within this pathway?

**Abstract:**

Lymphomas are a highly heterogeneous group of hematological neoplasms. Given their ethiopathogenic complexity, their classification and management can become difficult tasks; therefore, new approaches are continuously being sought. Metabolic reprogramming at the lipid level is a hot topic in cancer research, and sphingolipidomics has gained particular focus in this area due to the bioactive nature of molecules such as sphingoid bases, sphingosine-1-phosphate, ceramides, sphingomyelin, cerebrosides, globosides, and gangliosides. Sphingolipid metabolism has become especially exciting because they are involved in virtually every cellular process through an extremely intricate metabolic web; in fact, no two sphingolipids share the same fate. Unsurprisingly, a disruption at this level is a recurrent mechanism in lymphomagenesis, dissemination, and chemoresistance, which means potential biomarkers and therapeutical targets might be hiding within these pathways. Many comprehensive reviews describing their role in cancer exist, but because most research has been conducted in solid malignancies, evidence in lymphomagenesis is somewhat limited. In this review, we summarize key aspects of sphingolipid biochemistry and discuss their known impact in cancer biology, with a particular focus on lymphomas and possible therapeutical strategies against them.

## 1. Introduction

Metabolic reprogramming at the lipid level is one of the most relevant hallmarks of cancer. Although usually overshadowed by the more widely explored fields of oncogenomics and molecular biology, there are two main reasons to justify this statement. The first one is that the metabolome, especially the lipidome, represents a detailed phenotypic description of the neoplasm. In fact, its complexity rivals that of the proteome, and even the genome [1]. The second reason is the fact that lipid metabolites can potentially influence all other hallmarks of cancer at the genomic, transcriptomic, and proteomic levels through epigenetic changes, riboswitch regulation, and post-translational modifications, respectively [2,3]. Due to this, a great interest in studying lipid metabolism in cancer has emerged; however, this is not an easy task. Lipids are the most structurally diverse biomolecules, and cancer-related metabolic disruptions can be found in many lipid classes, including sterols, isoprenoids, acylglycerols, eicosanoids, phospholipids, and, of particular relevance, sphingolipids [4,5,6,7]. This last family has raised special attention due to their bioactive nature and their involvement in virtually every cellular function.

First discovered in 1876, sphingolipids received their name after the Sphinx, an enigmatic creature, due to their mysterious nature [8,9]. Their etymologic pertinence still applies to this day, since the more we learn about them, it becomes clearer that there is more to them than meets the eye. Considering that some, such as sphingosine-1-phosphate (S1P) and ceramide-1-phosphate (C1P), are bioactive soluble mediators, whereas others, such as sphingomyelin (SM) and glycosphingolipids (GSLs), are fundamental components of lipid rafts and glycosynapses, their functional relevance becomes evident [10,11,12]. They are involved in membrane transport, protein sorting, bioenergetics, vesicular trafficking, signal reception and transduction, gene expression and regulation, cell migration, cell cycle, senescence, and cell death, to mention just a few processes [1]. The issue, however, does not lie on the number of cellular processes that they mediate, but rather in the fact that each of them intervenes in different ways, creating a beautifully intricate web of divergent and redundant pathways, known as the sphingolipid rheostat [13].

**Figure 1:**
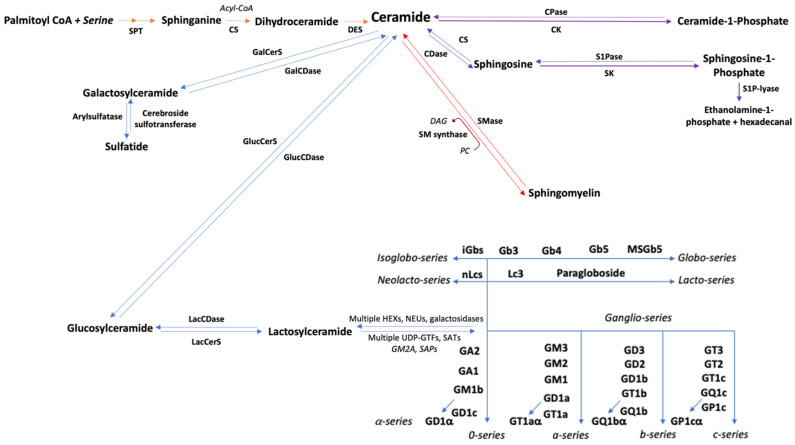
Overview of sphingolipid metabolism. SPT: serine palmitoyltransferase; CS: ceramide synthase; DES: dihydroceramide desaturase; CDase: ceramidase; SK: sphingosine kinase; S1Pase: sphingosine-1-phosphate-phosphatase; S1P-lyase: sphingosine-1-phosphate lyase; S1PR1: sphingosine-1-phosphate receptor 1; CK: ceramide kinase; CPase: ceramide-1-phosphate-phosphatase; SMS: sphingomyelin synthase; SMase: sphingomyelinase; PC: phosphocholine; DAG: diacylglycerol; GluCerS: glucosylceramide synthase; LacCerS: lactosylceramide synthase; HEXs: hexosaminidases; NEUs: neuraminidases; GTFs: glycosyltransferases; SATs: sialyltransferases; GM2A: ganglioside activator protein; SAPs: sphingolipid activator proteins (saposins); GlucCDase: glucosylceramidase; GalCerS: galactosylceramide synthase; GalCDase: galactosylceramidase. Considerations: Orange arrows depict de novo pathway. Purple arrows depict salvage pathway. Red arrows depict SM cycle. Blue arrows depict GSL metabolism. CDase and SMase have acid, alkaline, and neutral isotypes, depending on the subcellular compartment. Multiple intracellular transporters (CERT, FAPP2, CPTP, SPNS2, Mfsd2d, GLTP) move newly synthesized sphingolipids across subcellular compartments to ensure proper distribution.

Many aspects of their metabolism further emphasize sphingolipid complexity. As discussed later, ceramide is the central sphingolipid because it yields molecules that are in turn susceptible to a great plethora of modifications, such as phosphorylation, glycosylation, and sulfation (Figure 1) [14]. These processes might seem linear, but there are several factors that ultimately impact on the function of each specific species. The first one is the fact that there are many isotypes of the protein machinery (enzymes, intracellular transport proteins, and signaling receptors) needed for their biosynthesis, catabolism, and actions [15,16,17]. For instance, there are at least six ceramide synthases (CS), each of them codified by a different locus (*CERS1-6*), and each exhibiting preferential affinity towards certain fatty acid chains, therefore yielding different-sized ceramides which can have antagonic effects [18,19,20]. A second issue lies on the susceptibility of this same machinery to different post-translational modifications depending on tissular status, meaning that their action is potentially influenced by conditions such as infection, inflammation, and malignant transformation [21,22]. Considering these peculiarities, it is not surprising that sphingolipid research has gained relevance in different pathophysiological conditions, including autoimmunity, neurodegeneration, cardiometabolic disruption, and of course, carcinogenesis [23,24,25]. 

Cancer is among the leading causes of death in the world [26]. Although solid tumors currently hold the top spots for cancer incidence and mortality, hematologic malignancies such as leukemias, myelomas, and lymphomas are on the rise [27]. Out of these, lymphomas are the most prevalent in adults [26]. The term lymphoma encompasses an extremely diverse group of over 80 malignant blood disorders that arise from lymphocytes or their precursors. They can be broadly divided into Hodgkin’s lymphoma (HL) and Non-Hodgkin’s lymphoma (NHL), which as a group represents the most frequent hematologic neoplasm worldwide [26,28]. 

Since the introduction of anti-CD20 antibodies, the panorama of many B-cell lymphomas has significantly improved; however, some, such as diffuse large B-cell lymphoma (DLBCL) and mantle cell lymphoma (MCL), are inherently aggressive neoplasms with delicate prognoses even with proper treatment, which has prompted a never-ending quest for new therapeutic approaches [29,30]. Disease outcomes largely depend on the genomic background, and since the genetic heterogeneity of lymphomas is extreme, their clinical approach can become challenging. In fact, many cases remain in a classification gray zone even after immunophenotypic, genomic, and transcriptomic approaches [31,32,33]. Consequently, research has slowly been shifting towards the identification of extragenic parameters that could be involved in their onset, clinical course, and treatment response [34,35,36,37]. In recent years, clinical evidence has suggested that metabolic disturbances at the lipidic level might yield useful hints for risk stratification and treatment response, opening a whole new horizon for exploring the lipidomic reasoning behind these observations [38,39]. Given the fact that lipid metabolism is circular, and its pathways are deeply intertwined, deciphering the sphingolipidome has become especially appealing. This is a complex task, but understanding it is essential for the identification of new oncogenic pathways and chemoresistance mechanisms, and hence novel biomarkers and therapeutical targets. 

Extensive research regarding sphingolipid metabolism in cancer exists, but because most of it has been conducted in solid malignancies, evidence in lymphomagenesis is somewhat scarce. This is surprising when considering that many sphingolipids are powerful immune regulators whose hematopoietic-modulating properties, especially at the lymphopoietic level, have been well established [40,41,42,43,44,45,46,47,48]. Lymphomas have the peculiarity of being neoplasms of the immune system itself, meaning it is composed of cells that can innately circulate through the body, constantly interacting with lymphatic tissue that is physiologically suited to support their survival. Much of this stroma-tumor crosstalk is orchestrated by sphingolipid activity, which makes sense when considering that many genetic and chromosomic anomalies present in these neoplasms lead to sphingolipid-mediated cell survival. In fact, a recent genomic profiling study of T-cell lymphomas found sphingolipid signaling dysregulation to be one of the most prominent fingerprints [49,50,51]. The relevance of sphingolipids in this context is that, unlike other lipid biosignatures, and despite their complex behavior, their patterns seem to be conserved, even among malignancies as diverse as lymphomas [52,53]. In this review, we will summarize current knowledge on sphingolipid metabolism, from the simplest ones to the most complex, and their roles in cancer, with a particular focus on lymphomas. 

## 2. Sphingoid Bases

Sphingoid bases are the simplest sphingolipids. They include sphinganine (dihydrosphingosine), which is formed from the condensation of palmitoyl-CoA and serine, and sphingosine, which results from ceramide cleavage through a ceramidase [54]. Being the backbone of larger sphingolipids, their role in cancer has not received much attention; however, they are known to have antiproliferative properties by themselves, and can enhance chemosensitivity by inducing oxidative stress and upregulating p38 and JNK in TP53 positive lymphoma cells [55,56,57,58]. In DLBCL specifically, both sphingosine and sphinganine analogues induce cell death by promoting PARP cleavage, autophagy, and PKC inhibition [59,60]. 

## 3. Sphingosine-1-Phosphate

S1P is a metabolically active form of sphingosine which results from the action of a sphingosine kinase (SPHK) [1]. When it comes to cancer, it is probably the most widely recognized sphingolipid, and it is involved in the pathogenesis of multiple neoplasms, such as head and neck, breast, ovarian, colon, pancreatic, prostate, liver, and bile duct, among many others [61,62,63,64,65,66]. It is vigorously produced by both cancer cells and cells of the tumor microenvironment, such as tumor associated macrophages (TAMs), endothelium, and fibroblasts, creating a complex “inside-out” signaling hub which promotes invasion and metastasis due to its ability to induce cell proliferation and migration, angiogenesis, and tissue remodeling [67,68,69,70,71,72]. This is achieved through the upregulation of several proto-oncogenes, such as *MYC, FOS*, and *ABL1* [73,74,75,76]; extracellular matrix regulators, such as urokinase, Matrix Metalloprotease 2 (MMP-2), MMP-7, and syndecan-1 [77,78,79,80]; inflammatory mediators, such as IL-22 [81]; and transcription factors/transcriptional regulators, such as STAT3, MRTF-A, YAP, and SNAI2 [16,66,82]. Additionally, it favors CTFG and EGFR activation, which promote cancer cell motility through ezrin-radixin-moesin phosphorylation [83,84,85]. Moreover, it is known that autotaxin, which is upregulated in many cancers, generates many bioactive lipids, including lysophosphatidic acid (LPA) and S1P, which subsequently stimulate COX2 and therefore eicosanoid synthesis, yielding inflammatory conditions ideal for a tumorigenic microenvironment [18,21,86,87,88]. Furthermore, S1P pathways are related to chemotherapy resistance by upregulating the Multidrug Resistance gene (*MDR1*) [89]. This observation is consistent with the fact that SPHK1 activity and Sphingosine-1-Phosphate Receptor (S1PR) signaling appear to confer resistance to chemotherapy-induced apoptosis in many cancer models, whereas S1P-lyase, which mediates S1P degradation, has the opposite effect [90,91,92].

**Figure 2:**
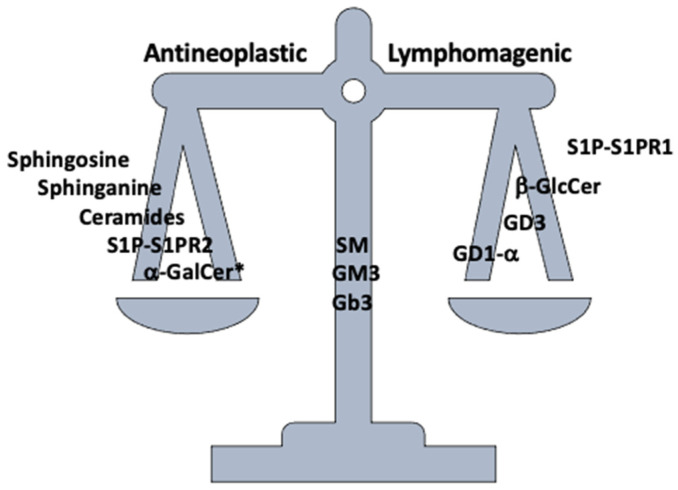
Summary of antineoplastic vs lymphomagenic sphingolipids. * Synthetic; S1P: Sphingosine-1-Phosphate; S1PR-2: Sphingosine-1-Phosphate Receptor 2; α-GalCer: α-galactosylceramide; SM: sphingomyelin; β-GlcCer: β-glucosylceramide; GM3: monosialodihexosylganglioside; Gb3: globotriaosylceramide.

As for lymphomas, SPHK overexpression has consistently been associated with a more aggressive disease [93,94,95]. In vitro evidence suggests that MCL cells can evade CD1d-mediated NKT cytotoxicity by upregulating SPHK1 [96,97]. Additionally, a recent report found that SPHK1 and S1P itself mediate a VEGF-independent mechanism of angiogenesis in DLBCL, which might explain why, although some preliminary data have suggested otherwise, classical anti-angiogenic drugs such as bevacizumab are mostly ineffective for NHL [98,99,100]. These data make SPHK an attractive therapeutical target. On the other hand, it has been shown that S1PR1 signaling promotes survival, proliferation, and migration of MCL and HL cells through a PI3K-dependent pathway, and might be useful as both a pharmacological target and an marker for aggressiveness [101,102,103]. In fact, tissular expression of S1PR1 has been associated with a worse prognosis in certain NHL, particularly primary testicular DLBCL [104,105]. Interestingly, immunohistochemical detection of different S1PR isotypes, migration integrins, chemokines, and homing receptors correlates with specific anatomical and tissular locations of B-cell lymphomas [106,107,108]. For instance, in MCL, *S1PR1* mutations are present in up to 8.6% of cases, and mediate tumoral cell retention in the mantle zone [109,110,111]. S1PR1 staining might be, in fact, a useful immunohistochemical marker for MCL, especially if cyclin D1 staining, the current standard, is inconclusive [112]. Additionally, it has been shown that mutations at this level (*S1PR1*) are partly responsible for the transformation of follicular lymphoma (FL) into its most aggressive form [113]. Contrastingly, S1PR2 activation shows completely opposite effects. Research suggests that it regulates cell survival and migration mainly through Akt and CXCL12 attenuation. In fact, the TGF-β/SMAD1/S1PR2 pathway is recurrently inactivated in DLBCL due to either disabling mutations in its axis or FOXP1-mediated downregulation [114,115,116]. Moreover, it has been recently reported that some EBV-related lymphomas downregulate S1PR2, allowing the PI3-K/Akt/mTOR pathway to be constitutively activated [17]. Naturally, while S1PR1 blockade is potentially antilymphomagenic, *S1PR2* deficient mice are prone to developing DLBCL [117,118,119]. These reports highlight the complex nature of sphingolipid-related molecular pathways, and stress the need to understand them (Figure 2). 

## 4. Ceramide

Ceramide is the central molecule of sphingolipid metabolism. It can be obtained by the hydrolysis of more complex sphingolipids, mainly SM via sphingomyelinase, or de novo through sphinganine fatty acylation and subsequent desaturation [54]. The length of the fatty acid chain has a relevant functional impact, being that C16, C18, and C24 are the most cytotoxic endogenously produced species. These sphingolipids are able to induce cell death through multiple pathways, including necroptosis, autophagy, mitophagy, necrosis, and especially apoptosis [120,121,122]. Caspase-dependent cell death mechanisms are achieved through Fas–FasL interaction, mitochondrial pore induction, TXNIP and BCLX upregulation, Rb overexpression, and telomere shortening via glyceraldehyde-3-phosphate dehydrogenase inhibition [123,124,125,126,127,128,129]. Furthermore, they also exhibit anti-proliferative effects through the activation of ceramide-activated protein phosphatases (CAPPs), which downregulate several CDKs; and PKC-ζ, which further leads to Akt attenuation [90,130]. This last observation is in line with the fact that ceramide and diacylglycerol (DAG), a potent PKC activator and thus pro-tumoral molecule, are simultaneously, but inversely, regulated during the SM cycle (Figure 1) [131]. Considering all of these potentially cytotoxic mechanisms, it is not surprising that many chemotherapeutic agents exert their effects partly through intracellular ceramide accumulation in the microenvironment, and within the tumor itself [132]. Unfortunately, cancer cells can develop resistance mechanisms against this pathway. Of note, it is known that acid ceramidase, which transforms ceramides back into sphingosine, is overexpressed in multiple cancers, such as head and neck, breast, prostate, melanoma, colon, glioblastoma, leukemia, and lung, where it promotes neosis, and mediates both chemoresistance and radioresistance [90,133,134,135,136,137,138,139,140,141]. Additionally, research suggests that the Ceramide Transfer Protein (CERT), which transports ceramide from the endoplasmic reticulum to the Golgi apparatus prior to its conversion to SM, might play a key role in antineoplastic resistance, as its downregulation has been shown to enhance chemosensitivity [142]. 

In the particular case of lymphomas, ceramides have been shown to contribute to IL-2 deprivation related cytotoxicity by degrading the apoptotic inhibitor IAP3 through cathepsin B in T-cell and NK lymphomas [143]. Additionally, it is known that some B-cell lymphomas can have mutations in *FVT1* (*KDSR*), a gene that codes for 3-ketodihydrosphingosine reductase, which synthesizes dehydrosphinganine, a precursor of ceramide. The fact that this locus is in close proximity to the much more recognized BCL-2 suggests a synergic role in lymphomagenesis [144]. Nonetheless, it is worth mentioning that different lymphomas have different patterns of *FVT1* alterations, therefore its metabolic implications are not the same. For instance, while some FL are known to overexpress FVT1, some DLBCL downregulate it. As a matter of fact, FVT1 expression might be useful to discriminate between germinal center (GC) DLBCL from non-GC DLBCL [145]. On a different note, blocking SPHK pathways, particularly via SPHK2, is cytotoxic to murine models of primary effusion lymphoma (PEL), a human herpesvirus 8-related neoplasm, due to the upregulation of ceramide synthase and subsequent accumulation of cytotoxic ceramide species that lead to apoptosis by viral lytic gene expression [146,147]. Additionally, ceramides have also been shown to induce cell death through caspase-independent mechanisms in MCL cells, possibly due to ROS associated necrosis and ATP depletion [148,149,150]. Similar effects have been observed after treatment with ceramide analogues and exogenous ceramide administration, which induce several tumor suppressor genes such as *CCL3*, *RHOB*, *KLF6*, and *THBS1* [151,152,153,154,155]. Finally, it is worth mentioning that rituximab, the cornerstone of B-cell NHL treatment, activates sphingomyelinase upon its binding to CD20, leading to an increased production of ceramide, and therefore selectively inducing cytotoxic pathways in CD20+ cells [156,157,158]. Similarly, it has been observed that newer anti-CD20 antibodies, such as tositumomab, are more effective in inducing programed cell death in NHL by inducing homotypic adhesion and lysosomal leakage, both of which are mediated by ceramide [143,159].

## 5. Ceramide-1-Phosphate

Similar to sphingosine, ceramide can also undergo phosphorylation by a ceramide kinase (CK) to produce ceramide-1-phosphate (C1P), whose functions are analogue to those of S1P and opposite to ceramide, meaning it is mainly involved in cell growth, migration, proliferation, and survival, all of which translate into cancer invasion and metastasis [54]. C1P is probably the least researched sphingolipid in the cancer context, with only a few studies linking it to neuroblastoma, and pancreatic and breast carcinomas [160]. Oncogenic and dissemination mechanisms include the PI3K/Akt/mTOR, MEK/ERK, and Rho/ROCK signaling pathways [161,162,163]. Although some of the initial studies describing the ceramide-C1P pathway were performed in leukemia cells, there is currently no published research linking C1P to lymphomas or any other hematological malignancy whatsoever [164].

## 6. Sphingomyelin

SM is a membrane sphingolipid that is abundantly found in membrane rafts, and thus is essential for signal transduction. In fact, K-Ras localization, and therefore MAPK/RAS signaling, is regulated by plasma membrane SM concentration [165,166]. SM results from ceramide condensation with phosphocholine via sphingomyelin synthase (SMS) [8]. As its biosynthesis requires ceramide metabolism, one might assume that these sphingolipids should have opposite effects. However, its actual role in cancer is controversial, as it is involved in both pro-tumoral and antineoplastic settings [167,168,169]. This duality might be partially explained by its central role in DAG/ceramide balance [170]. For instance, SMS2 has been shown to promote breast cancer metastasis by enhancing epithelial-to-mesenchymal transition (EMT) via TGF-β/Smad signaling [171]. On the other hand, SM levels seem to be inversely correlated with many cancer types, such as lung and esophageal [160,170]. Furthermore, exogenous SM administration promotes PPAR-γ mediated Th2 and anti-inflammatory responses, which are protective against cancer, and enhances chemotherapy-induced cytotoxicity by promoting drug influx and bioavailability [133,172,173,174].

In lymphomas most research points towards a primarily pro-tumorigenic effect. In vitro models have shown that SMS overexpression induces apoptosis resistance through PI3K-Akt upregulation, and stimulates malignant proliferation by promoting transferrin endocytosis in a SM-dependent manner [175]. These findings are in line with the fact that SMS inhibition or blockade enhances cell death due to ceramide accumulation, and inhibits infiltration by hindering the NF-κB pathway, subsequently downregulating adhesion molecules such as ICAM-1 [168,176]. On a different note, a recent clinical study found that even though total serum SM was similar between healthy controls and patients with hematologic malignancies, there were significantly lower levels of odd chain saturated fatty acids (OCFA) in the latter, which is interesting, since OCFA have recently been reported to be protective against several neoplasms, probably due to their histone deacetylase 6 inhibitor activity [177,178,179,180,181]. Finally, it has been observed that some lipid fragments of SM, together with specific phospholipid patterns, such as increased phosphatidylinositol and phosphatidylcholine, are markers of R-CHOP resistant or relapsed cases and of hypoxic and/or necrotic regions within the tumor [53]. These findings align with the previous observation that some endogenously produced phosphatidyl-myoinositols serve as physiologic inhibitors of sphingomyelinase [182]. 

## 7. Glycosphingolipids

GSLs are highly specialized, saccharide-containing sphingolipids that result from sequential glycosylation reactions from ceramide (Figure 1). This group includes cerebrosides, globosides, and gangliosides. GSLs are the core structures of glycosphingolipid enriched microdomains (GEMs) and glycosynapses, meaning they have a central role in signal recognition and transduction, and mediate complex cellular interactions [183]. Being such a large sphingolipid subfamily, GSLs’ effects on cancer can be strikingly diverse, and have been extensively researched in this area (Figure 2 and Table 1) [184,185]. It is known that many GSLs are differentially found in several solid malignancies, such as cholangiocarcinoma and ovarian cancer, and some of them have even been proposed as potential biomarkers for these tumors [186,187,188,189,190]. This differential expression, along with the fact that during malignant transformation they undergo cancer-specific modifications such as fucosylation, has allowed for the development of promising targeted immunotherapies, including monoclonal antibodies, vaccines, and CAR-T cells against aggressive malignancies such as neuroblastoma, retinoblastoma, and Ewing’s sarcoma [191,192,193,194,195,196].

### 7.1. Cerebrosides

Cerebrosides contain a single monosaccharide moiety as a side chain. This sugar can be either glucose or galactose, which helps to further subdivide this family into glucocerebrosides and galactocerebrosides. This latter group can undergo additional sulfuric esterification thanks to a cerebroside sulfotransferase, yielding metabolically active compounds known as sulfatides [18]. One of the main chemoresistance mechanisms in cancer is ceramide activity neutralization through its metabolism into hexosylceramides, mainly glucosylceramide (GlcCer). Multiple studies have shown that this cerebroside upregulates the multidrug efflux pump P-glycoprotein, stimulates Akt and survivin pathways, and blocks NADPH oxidase activity, and thus oxidative stress-induced cell death [203,204,205,206,207]. Both GlcCer synthase and P-glycoprotein expression have in fact been associated with lymphovascular invasion in oral cavity cancer [208]. In line with these findings, it has been shown that GlcCer synthase suppression restores p53 function, and enhances chemosensitivity in head and neck neoplasms [209,210]. Similarly, galactosylceramide and sulfatide also exhibit pro-tumorigenic properties in different cancer cells, such as breast and liver, by inhibiting apoptosis, increasing P-selectin expression, and promoting cell migration [211,212,213]. Several synthetic cerebrosides, however, stimulate anti-tumor immunosurveillance, and have been extensively researched as part of novel antineoplastic strategies against various cancers, including colon, ovarian, brain, and melanoma [214,215,216,217].

A clear example of how GSL metabolism disruption at this level may culminate in lymphomagenesis is Gaucher’s disease, a β-glucocerebrosidase deficiency that conditions an abnormal accumulation of GlcCer in blood cells and other tissues. This sphingolipidosis is associated with a high risk of developing aggressive hematologic malignancies, particularly NHL. Although the underlying biochemical mechanisms are still unclear, research has shown that GlcCer synthase inhibition significantly reduces such risk [218,219,220]. Contrastingly, dendritic cell vaccines combined with the synthetic α-galactosylceramide has shown promising results in B-cell lymphoma models due to its ability to induce strong CD1d-mediated iNKT [221,222]. This strategy seems to confer robust, long-lasting anti-tumor responses through both IFN-γ induction and adaptative immunity stimulation [199].

### 7.2. Globosides

Globosides are more complex sphingolipids that contain an oligosaccharide side chain, which is formed by different combinations of glucose, galactose, and N-acetylgalactosamine. They serve as bacterial toxin receptors, and are essential for cell phenotyping. For instance, Gb3 (CD77) is mainly expressed on the surface of different epithelia, where they bind Shiga toxins produced by *Shigella* spp. and *E. coli* spp. prior to their endocytosis [223]. Additionally, the P1PK, ABH and Lewis blood group systems are based on glycosylic modifications of globosidic antigens [183,224]. These GSLs have been associated with primarily pro-tumorigenic effects in many cancers, including gastric, breast, mesothelioma, prostate, liver, bile duct, colon, breast, and thyroid [225,226,227,228,229,230,231,232,233]. Gb4, for instance, is able to stimulate cell growth and proliferation due to its well-known ability to activate the EGFR/MAPK/ERK pathway [234]. Furthermore, Gb3 (CD77) and Gb4, similarly to GlcCer, have been reported to induce MDR1 expression and the mutant version of p53 through c-Src kinase recruitment and the nuclear translocation of β-catenin [207,235,236]. Longer globosides such as Gb5 (SSEA3), MSGb5 (SSEA4), and Globo-H, which is exclusively expressed in cancer cells, are associated with anaplasia, stemness, EMT, anoikis resistance, and metastasis [225,226,227,228,229,230]. Some of the mechanisms that explain these hostile properties include FAK/CAV1/AKT/RIP complex stimulation, MMP-2 and MMP-9 induction, integrin upregulation, and TRAX-dependent angiogenesis [237,238,239,240]. 

Although globoside research in lymphomas is limited, it has been repeatedly observed that Gb3 directly regulates the expression and availability of different plasma membrane receptors, such as CD20, CD19, and IFN-α receptor in B-cell lymphoma cells due to the presence of Gb3 binding sites in said proteins [241,242,243,244,245]. These findings suggests that globoside concentration in the lipid bilayer might be involved in immunotherapy response, IFN-mediated growth inhibition, and antigenic escape. Similarly, this globoside has also been shown to regulate downstream BCR signaling in Burkitt lymphoma (BL) cells by activating Lyn and Syk kinases, suggesting that it is also involved in BCR-mediated apoptosis regulation. [246]. Additionally, as mentioned previously, Gb3 serves as a bacterial toxin receptor, which has prompted the development of engineered toxin bodies (ETBs) in order to modify and reposition these toxins as antitumor tools [223,247,248,249,250,251,252].

### 7.3. Gangliosides

Gangliosides are the most complex GSLs and are distinguished from globosides due to the presence of at least one n-acetylneuraminic acid (NANA) molecule, which is the predominant sialic acid form found in humans. They serve as glycoproteic molecule receptors, and are essential in phenotyping and differentiation. They can be further subdivided in monosialylgangliosides (GM), disyalogangliosides (GD), trisyalogangliosides (GT), and tetrasyalogangliosides (GQ), depending on the number of NANA residues which are present in the molecule [253]. These residues allow them to bind to different types of tissues, which is why they have a predominant role in cell migration, angiogenesis, and immune regulation. In fact, it is known that GM1-enriched lipid rafts are crucial for BCR signaling upon B-cell activation [254]. Their powerful immunogenicity is evidenced by the fact that many autoimmune disorders and paraneoplastic syndromes, some of which can occur in B-cell neoplasms, occur due to anti-ganglioside autoantibodies [255]. Unsurprisingly, their role in cancer biology is very complex [256]. In fact, most of them exhibit both pro-tumorigenic and antineoplastic properties, depending on cancer type and stage. Remarkably, this duality might be mediated by abnormal glycosylation patterns that occur specifically in malignantly transformed cells [257]. 

Some GMs, such as GM1 and GM3, exhibit predominantly anti-proliferative effects in different cancers, such as colon, bladder, gliomas, and leukemias, by modulating cell cycle progression through PTEN and p53 stimulation, mitigating PDGF-mediated MAPK activation, and promoting apoptosis through BAX and BAD upregulation [235,258,259,260,261]. Furthermore, GM3 and GM1 expression have consistently been reported to weaken metastatic potential in gastrointestinal and ovarian carcinomas by inhibiting cell motility and MMP-9-mediated migration [262,263,264]. Interestingly, GM3 is also able to induce MMP-2-mediated invasion of melanoma, and positively correlates with Ki-67 status in breast cancer, which is puzzling when considering that GM3 synthase silencing has been shown to decrease breast cancer metastases in vivo via NFAT1 inhibition [265,266,267,268,269]. Similarly, GM3 enhances sensitivity to EGFR-TK inhibitors in lung cancer while promoting resistance to classic chemotherapeutic agents in this same neoplasm, suggesting it has divergent roles within specific cellular pathways, such as apoptosis [270,271]. As for disyalogangliosides, GD1a seems to be linked to a decreased metastatic potential in renal carcinoma and osteosarcoma cells through MMP1 and MMP7 downregulation, EGFR and FAK/Akt signaling inhibition, β1 integrin recycling blockage and degradation, and HGF-mediated motility decline [272,273]. Surprisingly, this ganglioside is also associated with caveolin-1 and STIM-1 expression, which means its antimetastatic potential is debatable at best [273,274,275]. Finally, GD3, which is abundantly found in neuroectoderm-derived tumors, behaves in a similar way [235]. On the one hand, it induces apoptosis and proliferation arrest through mitochondrial permeabilization, ROS generation, EGF and VEGF inhibition, β1-integrin mediated anchorage disruption, and c-Src/NF-kB dismantling [276,277,278,279]. On the other hand, its role in promoting stemness and survival through SNAI1, TWIST1, TGF-β, c-Met, Akt, and ERK has been well documented [280,281]. 

It is known that some gangliosides, such as GM2, are actively shed from lymphoma cells into the microenvironment, where they modulate growth factor signaling and exert immunosuppressive effects to inhibit NK-mediated lysis [282,283]. Additionally, in B-cell lymphomas, GM3-enriched GEMs are essential to improve tumor necrosis factor-related apoptosis-inducing ligand (TRAIL) selective binding to its death receptor (DR4) in malignant cells [284]. GM3 has in fact been recently proposed as a useful serum biomarker for discriminating lymphoid neoplasms from healthy plasma [285]. The fact that GM3 has consistently been associated with lymphomagenesis supports the observation that hexosaminidase, the enzyme responsible of metabolizing GM2 into GM3, could be used as a clinical biomarker for several lymphomas [286,287,288]. Furthermore, GM1 concentration has been found to be directly associated with rituximab response in DLBCL, BL, and MCL [289]. Moreover, a recent study found that GD3, which is overexpressed on the surface of cutaneous T-cell lymphoma cells (CTCL), inhibits IL-17 production from healthy CD4^+^ T-cells in the tumor microenvironment, compromising cancer immunosurveillance, thus allowing malignant cells to proliferate unsuppressed [202]. Finally, it is worth mentioning that ceramide cytotoxicity seems to be inversely correlated to anaplastic lymphoma cell surface sialylation, whereas cell adhesion and invasion seems to have the opposite tendency [290,291,292,293]. These reports are interesting because the amount of sialylation directly depends on ganglioside content, and poses the question of researching the therapeutic potential of neuraminidase in this context. 

## 8. Targeting Sphingolipid Metabolism in Lymphoma Treatment

**Figure 3:**
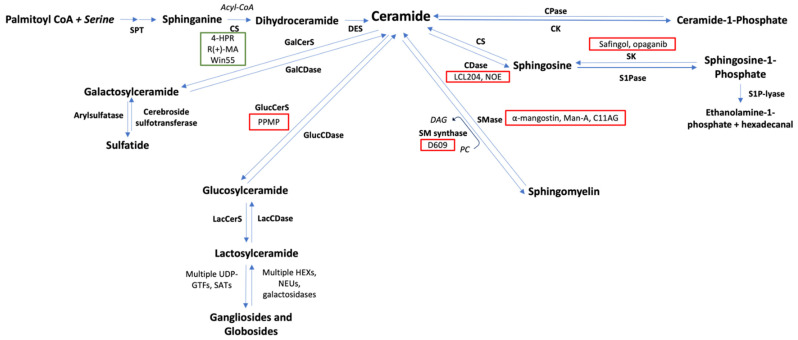
Potential therapeutical with published evidence against lymphomas. Green boxes represent enzymatic inducers and red boxers represent enzymatic inhibitors. SPT: serine palmitoyltransferase; CS: ceramide synthase; DES: dihydroceramide desaturase; CDase: ceramidase; SK: sphingosine kinase; S1Pase: sphingosine-1-phosphate-phosphatase; S1P-lyase: sphingosine-1-phosphate lyase; S1PR1: sphingosine-1-phosphate receptor 1; CK: ceramide kinase; CPase: ceramide-1-phosphate-phosphatase; SMS: sphingomyelin synthase; SMase: sphingomyelinase; PC: phosphocholine; DAG: diacylglycerol; GluCerS: glucosylceramide synthase; LacCerS: lactosylceramide synthase; HEXs: hexosaminidases; NEUs: neuraminidases; GTFs: glycosyltransferases; SATs: sialyltransferases; GlucCDase: glucosylceramidase; GalCerS: galactosylceramide synthase; GalCDase: galactosylceramidase; 4-HPRT: N-(4-hydroxypheny) retinamide (fenretinide); R(+)-MA: R(+)-methanandamide; PPMP: 1-phenyl-2-palmitoylamino-3-morpholino-1-propanol; D609: Tricyclodecan-9-yl-xanthogenate; NOE: N-oleoylethanolamine; Man-A: Manomycin A; C11AG: undecylidene-aminoguanidine.

As we have discussed this far, the role of sphingolipids in lymphomagenesis is becoming overwhelmingly recognized. This evidence, together with the fact that most of the chemotherapeutic agents classically used against lymphomas exert their cytotoxicity partially through ceramide accumulation, has fueled research on the therapeutic potential of sphingolipid metabolism [132,294,295]. Since these molecules are involved in many aspects of lymphomagenesis, targeting them is indeed a smart strategy; however, carefully designed approaches are imperative. When analyzing sphingolipid pathways and their biological effects, many possible targets stand out at first glance; however, this does not necessarily mean that they are all equally feasible. In the last decade, hundreds of sphingolipid metabolism-modifying substances have been identified. Many of them are naturally occurring fungal or bacterial toxins and plant derivatives, but most of them are synthetic chemicals. Remarkably, many FDA approved drugs, such as tricyclic antidepressants, antipsychotics, COX-2 inhibitors, and bisphosphonates, have been reported to alter sphingolipid metabolism at different levels [296,297,298,299,300,301]. Naturally, most of these substances have been tested as anti-cancer agents, out of which only around 10 have shown efficacy against lymphomas in preclinical models. Unfortunately, only a handful of them have reached clinical trials (Figure 3). 

Logically, most approaches have intended to take advantage of ceramide cytotoxicity through exogenous ceramide administration (ceramide analogues or synthetic ceramides) or by promoting ceramide buildup. For instance, it has been shown that treatment with short-chained ceramides, such as C2-cer and C6-cer, induces paraptosis and apoptosis in BL and Fas-resistant HL cells, respectively [302,303]. C6-cer has in fact been recently proposed as a promising treatment against mycosis fungoides and Sézary syndrome, currently uncurable forms of cutaneous T-cell lymphomas. This group demonstrated that C6-cer was selectively toxic towards malignant cells due to a relative ceramidase deficiency as compared to keratinocytes [153]. Current approaches for optimizing ceramide-based treatments include nanoliposome preparations and chemical modifications to render cationic species with a higher specificity for the negatively charged mitochondrion [301]. Sadly, none of these approaches have reached clinical trials in the context of lymphomas.

Similarly, some substances promote CS transcription and activity. For instance, some cannabinoid analogues, such as R(+)-methanandamide and Win55, promote apoptosis in MCL cells via CS upregulation and p38 phosphorylation [149,304]. Likewise, retinoids, which are vitamin A-derived molecules, are known to strongly upregulate CS as well. Some of them have long been used against certain hematologic malignancies, mainly acute promyelocytic leukemia, due to their ability to induce blast differentiation; however, their role in mature blood neoplasms, including lymphomas, is increasingly being recognized due to their ceramide-related cytotoxic properties [305,306,307,308,309]. The most successful case is fenretinide (4-HPR). This synthetic retinoid strongly promotes ceramide accumulation, mitochondrial depolarization, BAX translocation, caspase-activation, ROS generation, and IκBα kinase downregulation in many lymphoma cell models [310,311,312,313,314]. Fenretinide has also shown preclinical synergism with many classes of anti-lymphoma agents, such as histone-deacetylase inhibitors (vorinostat), proteosome inhibitors (bortezomib), and, of course, rituximab, which has led to its evaluation in phase I-II trials, yielding overall modest responses [313,315,316,317,318]. 

Ceramidase inhibition is another potential way of promoting ceramide buildup; however, although existing ceramidase inhibitors such as ceranib-1 and ceranib-2 have clearly shown anticancer effects in vitro, little evidence exists regarding their use in lymphomas [319,320,321]. Alternatively, ceramide accumulation can be achieved by inhibiting SM synthesis. In fact, SMS inhibitor tricyclodecan-9-yl-xanthogenate (D609) was found to be cytotoxic against methotrexate-resistant murine chronic lymphocytic leukemia (CLL) in vitro, probably due to upstream ceramide accumulation [322,323]. Nonetheless, as previously discussed, SM is a complex molecule, and its role in lymphomagenesis is still uncertain, so much so that SMase inhibitors, such as undecylidene-aminoguanidine (C11AG), manomycin A, and α-mangostin, have also shown to be cytotoxic to many lymphoma cell lines [324,325]. The reality is that these strategies are impractical for several reasons. To begin with, SM is an essential constituent of membrane rafts, and therefore influences cellular dynamics at multiple levels. For instance, its concentration in the plasma membrane is critical for endocytosing alkylphospholipids and other antineoplastic drugs used against lymphomas [201,326]. Additionally, some of the previously mentioned SMase inhibitors are not selective, and impact lipid metabolism at other levels. For instance, D609 is a well-known phospholipase C inhibitor, meaning it does not only regulate ceramide concentration, but also hinders potentially pro-tumorigenic DAG-mediated signaling [322]. Similarly, manomycin A is also known to inhibit farnesyltransferase, whose activity is necessary for Ras GTPase prenylation, and is frequently overexpressed in cancer, making it hard to pinpoint its tumoricidal properties to SMase inhibition [327,328,329,330]. In the same manner, LCL204, one of the only acid ceramidase inhibitors tested in this context, also halts N-myristoyltransferase activity, which is also essential for lymphomagenesis [319,331]. All of these off-target effects, together with the fact that clinical evidence is lacking, make these molecules overall weak candidates for lymphoma treatment.

The S1P pathway, on the other hand, is a much more plausible target, but some aspects still need thorough consideration. Many SPHK inhibitors have been described, but once again, their evidence regarding lymphomas is limited [332,333,334]. Safingol, a SPHK1 inhibitor, has shown efficacy in preclinical models; however, the first and only phase I trial that intended to evaluate a combination of fenretinide and safingol against relapsed malignancies (both solid and NHL) was terminated due to logistical issues (Clinical Trial Identifier: NCT01553071). It is worth mentioning that, similar to SMase inhibitors, this drug is nonspecific and also targets PKC, meaning it has multiple antineoplastic mechanisms [335,336]. Selective SPHK2 inhibitors have also shown great potential for cancer treatment in vitro [155,337]. Particularly, Opaganib (ABC294640) has shown promise in the treatment of liver, pancreatic, and bile duct tumors. Sadly, a phase I-IIa trial that intended to evaluate it in relapsed DLBCL was discontinued due to a lack of recruitment (Clinical Trial Identifier: NCT02229981).

Several therapeutic antibodies that modulate this pathway exist. For instance, sonepcizumab, a S1P neutralizing antibody, has previously been evaluated in the context of solid malignancies, but its efficacy in lymphoma is lacking [332]. Nevertheless, blocking S1P synthesis and/or action altogether would not be wise. It is imperative to bear in mind that at least five subtypes of the S1PR exist, all of which are activated by S1P, but whose effects can be completely antagonistic. Such is the case of S1PR1, which has overwhelmingly lymphomagenic properties, and S1PR2, whose activation has a predominantly tumor suppressive role [338,339]. S1PR1 has in fact consistently demonstrated to downregulate MyD88/JAK/STAT3 activation, and therefore survival signals such as Akt and IL-6 in Activated B-Cell (ABC) DLBCL ex vivo [340,341]. In recent years, the use of fingolimod, a S1PR modulator, as a potential cancer treatment has arisen [342]. In the case of lymphomas, this sounds particularly attractive due to the theoretical ability of this drug to sequester circulating malignant lymphocytes into the secondary lymphoid organs, hence preventing them from spreading [343]. However, it is essential to consider that this drug is an immunosuppressant. In fact, it is currently only approved for autoimmune disorders such as multiple sclerosis, and therefore using it in the context of lymphoma, an already immunosuppressing condition, would be imprudent. In fact, there are several reports of lymphoproliferative disorders as a secondary effect of fingolimod, probably due to immunosurveillance disruption, which is remarkable when considering that it binds to all S1PR isotypes except for S1PR2 [344,345]. Novel S1PR modulators, such as siponimod, ozanimod, ponesimod, ceralifimod, and amiselimod, have different affinities for the various S1PR subtypes, but these drugs are relatively new, and are currently only being researched in the context of autoimmunity [346]. Additional experimental evidence against cancer and longitudinal studies assessing the incidence of lymphomas among users of these medications are necessary before considering further clinical evaluation in this context. 

GSLs are the most complex sphingolipids, and thus their effects on cancer are the most difficult to predict. These molecules do not have the clearly defined effects of their smaller cousins. There are no universally mitogenic or cytotoxic GSLs, but some tendencies can be observed. For instance, as previously discussed, the risk of developing NHL in Gaucher’s disease is clearly higher, posing the question of the role of glucocerebrosides in lymphomagenesis [295,347]. Miglustat and eliglustat are substrate reduction therapy drugs that competitively inhibit glucosylceramide synthase. Some preliminary preclinical evidence suggests that these molecules could have antitumoral effects, but being relatively new, there is currently no clinical research of these drugs in a context different than lysosomal storage diseases [218,348]. Similarly, imiglucerase, velaglucerase, and talilglucerase are clinically approved enzyme replacement therapy drugs that hydrolyze glucocerebroside into glucose and ceramide [349]. Although their role in cancer has not been researched, the rationale is correct, and it would be interesting to evaluate them in lymphomas.

The fact that GSLs are cell surface markers is bittersweet, because it makes them technically easy to target through immunotherapeutic approaches; however, migrating them to a clinical setting would probably not be as straightforward. First of all, as we have discussed, these molecules exhibit a great duality in cancer, and predicting their effects in malignancies as diverse as lymphomas would be problematic. Additionally, the fact that their metabolism occurs in a cascade-like fashion poses the risk that inhibiting the synthesis of theoretically lymphomagenic species could cause a paradoxical upstream accumulation of potentially tumorigenic precursors (Figure 1). The chances of this occurring would decrease further down the pathway, meaning that blocking alpha-series gangliosides such as GD1α could be reasonable, especially considering its role as a metastasis promoter in certain murine lymphomas cell lines [350]. According to NIH information, tens of therapeutic anti-ganglioside antibodies are currently being researched in the context of solid malignancies such as bone sarcomas and central nervous system tumors. Some of these agents include racotumomab (anti-GM3), ecromeximab (anti-GD3), dinutuximab (anti-GD2), naxitamab (anti-GD2), nivatrotamab (bispecific anti-GD2xCD3), and multiple polyvalent ganglioside vaccines [351]. None of these approaches have been clinically evaluated against lymphomas; however, anti-GD2 monoclonal antibodies have successfully induced lymphoma cell death in vitro [352].

## 9. Concluding Remarks and Future Perspectives

Aiming for sphingolipid pathways is an appealing strategy for lymphoma management; however, given the complexity of their metabolism, precise interventions are crucial. As we have thoroughly discussed, sphingolipids can exhibit different properties depending on multiple factors, such as the genetic background and microenvironment; consequently, while useful, targeting these pathways is far from being a medical panacea. In fact, it is quite possible that what works for certain circumstances will worsen others, despite pathophysiological proximity. This is particularly true for lymphomas, since even if they are clustered within the same category, their etiological heterogeneity makes each case unique. Let us also remember that sphingolipids are involved in every aspect of cell biology; therefore, assuming that manipulating a specific biochemical pathway would only have a handful of predictable effects is a mistake. These factors might seem impractical and discouraging, but they represent a great area of opportunity. The lipidome accurately depicts what is really going on inside the cell regardless of the underlying genome, transcriptome, or proteome, which allows for the identification of exciting theranostic biomarkers that could bring precision healthcare closer to lymphomas. Integrative multiomic approaches are the next step to overcome the gap between lab findings and their actual clinical translation; and even if unraveling the mysteries of sphingolipid biology continues to render perplexing clues that emphasize their dichotomous nature, we must learn to take advantage of both edges of this sword in our favor.

## Figures and Tables

**Table 1:** Role and possible mechanisms of selected sphingolipids in lymphomagenesis.

Sphingolipid	Possible Mechanism	Reference
Predominantly Antineoplastic		
Sphingoid bases	↑ ROS ↑ p38, JNK ↑ cPARP ↑ AIF, Bak ↑ Lck ↓ PKC	[55,56,59,197,198]
Sphingosine-1-phosphate (S1PR2)	↓ CXCL12 mediated migration ↓ PI3K-Akt-mTOR ↑ TGF-β/SMAD1/S1PR2	[17,114,117]
Ceramide	↑ Cytochrome c release ↑ Fas/FasL ↑ ROS ↑ *CCL3, RHOB, KLF6, THBS1* ↑ JNK, ERK, p38, p21, p27 ↓ IAP3 ↓ PKC	[123,124,143,150,156]
α-galactosylceramide *	↑ CD1d-mediated immune cytotoxicity	[199]
Predominantly Lymphomagenic		
Sphingosine-1-phosphate (S1PR1)	↓ CD1d-mediated immune cytotoxicity ↑ VEGF-independent angiogenesis ↑ PI3K mediated migration	[96,98,200]
Sphingomyelin	↑ PI3K-Akt ↑ NFκB ↑ ICAM ↑ Transferrin endocytosis	[22,175,176,201]
GD3	↓ IL-17	[202]

* Synthetic; ROS: reactive oxygen species; JNK: c-JUN N-terminal kinase; cPARP: cleaved Poly ADP Ribose Polymerase; AIF: apoptosis-inducing factor; PKC: protein kinase C; S1PR: sphingosine-1-phosphate receptor; ROCK: Rho-associated protein kinase; PI3K: phosphatidylinositol 3-kinase; IAP3: inhibitor of apoptosis protein 3; VEGF: vascular endothelial growth factor; ICAM: intercellular adhesion molecule 1; IL-17: interleukin 17.
